# Phenolic Compositions and Antioxidant Properties in Bark, Flower, Inner Skin, Kernel and Leaf Extracts of *Castanea crenata* Sieb. et Zucc

**DOI:** 10.3390/antiox6020031

**Published:** 2017-05-05

**Authors:** Phung Thi Tuyen, Tran Dang Xuan, Do Tan Khang, Ateeque Ahmad, Nguyen Van Quan, Truong Thi Tu Anh, La Hoang Anh, Truong Ngoc Minh

**Affiliations:** 1 Graduate School for International Development and Cooperation, Hiroshima University, Higashi-Hiroshima city, Hiroshima 739-8529, Japan; phungtuyen@gmail.com (P.T.T.); dtkhang@ctu.edu.vn (D.T.K.); nguyenquan26@gmail.com (N.V.Q.); tuanhbp@gmail.com (T.T.T.A.); hoanganh6920@gmail.com (L.H.A.); minhtn689@gmail.com (T.N.M.); 2 Process Chemistry and Technology Department, CSIR-Central Institute of Medicinal and Aromatic Plants, Lucknow 226015, India; ateeque97@gmail.com

**Keywords:** *Castanea crenata*, inner skins, phenolics, flavonoids, column chromatography, ellagic acid, myricetin

## Abstract

In this study, different plant parts (barks, flowers, inner skins, kernels and leaves) of *Castanea crenata* (Japanese chestnut) were analyzed for total phenolic, flavonoid, and tannin contents. Antioxidant properties were evaluated by using 1,1-diphenyl-2-picrylhydrazyl (DPPH), 2,2′-azinobis (3-ethylbenzothiazoline-6-sulfonic acid) diammonium salt (ABTS), reducing power, and β-carotene bleaching methods. The highest total phenolic and tannin contents were found in the inner skins (1034 ± 7.21 mg gallic acid equivalent/g extract and 253.89 ± 5.59 mg catechin equivalent/g extract, respectively). The maximum total flavonoid content was observed in the flowers (147.41 ± 1.61 mg rutin equivalent/g extract). The inner skins showed the strongest antioxidant activities in all evaluated assays. Thirteen phenolic acids and eight flavonoids were detected and quantified for the first time. Major phenolic acids were gallic, ellagic, sinapic, and *p*-coumaric acids, while the principal flavonoids were myricetin and isoquercitrin. The inner skin extract was further fractionated by column chromatography to yield four fractions, of which fraction F3 exhibited the most remarkable DPPH scavenging capacity. These results suggest that *C. crenata* provides promising antioxidant capacities, and is a potential natural preservative agent in food and pharmaceutical industries.

## 1. Introduction 

*Castanea crenata* Sieb. et Zucc (Japanese chestnut), belonging to the family Fagaceae, is a woody native plant of Japan and South Korea, and widely cultivated in Asian countries [1,2]. The total annual chestnut consumption in Japan is above 20,000 tons [3]. This species has been used as a traditional medicine in many countries for centuries [4]. Particularly, its leaves have been applied in treatment of whooping cough and lacquer poisoning in folk medicine [2]. Furthermore, the inner skins of chestnuts have long been used in East Asia, especially in Korea, as an ingredient in cosmetics [5].

Among more than 25,000 secondary metabolites that have been identified in plants [6], phenolic compounds have been exploited as scavengers and inhibitors due to their antioxidant, antibacterial, anti-allergic, anti-inflammatory, anti-aging, and anti-tumor properties [7,8]. Therefore, phenolic compounds are increasingly applied in food, pharmaceutical and cosmetic productions [9].

Many studies have been extensively conducted in recent years to identify and quantify phenolic compounds and the relevant antioxidant activity of plants [10], and *C. crenata* has attracted much attention because of its high potential biological activities. In a study by Carocho et al. [11], the extracts of chestnut skins showed strong antioxidant activities, and contained a large quantity of polyphenols, especially condensed and hydrolysable tannins [10,12,13]. Kurigalin, a agallotannin, was isolated from barks of *C. crenata* [14]. Other studies noted that bark extracts contained high concentrations of ellagic and gallic acids [15]. However, no available data on the fractionation and quantification of phenolic components of extracts from different aerial parts of *C. crenata* are reported. In this paper, free and bound phenolics obtained from bark, flower, inner skin, kernel, and leaf extracts of *C. crenata* were identified and quantified. In addition, the crude and fractionated extracts from column chromatography were evaluated for the antioxidant activities. The correlation between phenolic contents and antioxidant capacities was also investigated.

## 2. Materials and Methods

### 2.1. Standards and Reagents

Standard compounds including gallic acid, protocatechuic acid, catechol, chlorogenic acid, *p*-hydroxybenzoic acid, vanillic acid, caffeic acid, sinapic acid, syringic acid, vanillin, ferulic acid, *p*-coumaric acid, benzoic acid, ellagic acid, cinnamic acid, apigenin, (+)-catechin, esculetin, fisetin, galangin, isohamnetin, isoquercitrin, kaempferol, luteolin, myricetin, quercetin, rhamnetin, and rutin, were purchased from Kanto Chemical Co., Inc., Tokyo, Japan.

Reagents including 1,1-diphenyl-2-picrylhydrazyl (DPPH), dibutyl hydroxytoluene (BHT), 2,2′-azinobis (3-ethylbenzothiazoline-6-sulfonic acid) diammonium salt (ABTS), potassium persulfate (K_2_S_2_O_8_), Folin-Ciocalteu’s reagent, aluminium (III) chloride hexahydrate (AlCl_3_·6H_2_O), β-carotene, linoleic acid, polyoxyethylen esorbitan monopalmitate (Tween-40), trichloroacetic acid (CCl_3_COOH), iron (III) chloride (FeCl_3_), sodium carbonate (Na_2_CO_3_), disodium hydrogenphosphate (Na_2_HPO_4_), sodium dihydrogenphosphate (NaH_2_PO_4_), sodium hydroxide (NaOH), and hydrochloric acid (HCl), were obtained from Kanto Chemical Co., Inc., Tokyo, Japan. All chemicals used were of analytical grade.

### 2.2. Plant Materials

Barks, leaves, flowers, and nuts from *Castanea crenata* trees were collected from Mt. Takanosu and Ikoinomori park (Hiroshima, Japan) in May and October 2015. The voucher specimens (IK23520151 and IK23520152) have been deposited at the Laboratory of Plant Physiology and Biochemistry, Graduate School for International Development and Cooperation (IDEC), Hiroshima University, Higashi-Hiroshima city, Japan. The nuts were separated by hand into kernels and inner skins. All the samples were dried in an oven at 30 °C for one week, and then pulverized into fine powder.

### 2.3. Preparation of Extracts

Free phenolics were extracted by stirring dried powder (6 g) in 200 mL ethanol (100%) for 24 h at room temperature. Solvent was removed by a rotary evaporator (SB-350-EYELA, Tokyo, Japan) at 30 °C. The precipitates were weighed, dissolved in methanol, and kept in the dark at 4 °C for further analysis. Bound phenolics were extracted from the residues of free phenolic extraction by hydrolyzing with 100 mL of 4 M NaOH, and stirring for 4 h at 50 °C. This suspension was filtered and the pH was adjusted to pH 1.5 with 37% hydrochloric acid. The filtrate was extracted three times with ethyl acetate. Finally, the extracts were combined and evaporated to dryness in a rotary evaporator at 30 °C, and dissolved in methanol to obtain stock concentrations of 10 mg/mL, and then stored in the dark at 4 °C for further analysis.

### 2.4. Determination of Total Phenolic Contents

Phenolic content was estimated following the Folin-Cicalteau method described by Medini et al. [16] with minor modifications. The extract concentrations ranged from 100 to 500 µg/mL. The total phenolic contents were expressed as mg gallic acid equivalent (GAE)/g extract.

### 2.5. Determination of Total Flavonoid Contents

Total flavonoid contents were evaluated using the aluminum chloride colorimetric method described by Bueno-Costa et al. [17]. A volume of extract (0.5 mL) was mixed with 2% aluminum chloride solution (0.5 mL). The mixture was kept for 15 min at room temperature, and the absorption was measured at 430 nm. The total flavonoid contents were expressed as mg rutin equivalent (RE)/g extract.

### 2.6. Determination of Total Tannin Contents

Total tannin contents were measured by the vanillin/HCl method of Rebaya et al. [18] with minor modifications. A volume of 0.4 mL of extract was added to 3 mL of vanillin (4% in methanol) and 1.5 mL of concentrated hydrochloric acid. After 15 min of incubation, the absorbance was read at 500 nm. The amounts of total tannin were expressed as mg (+)-catechin equivalent (CE)/g extract.

### 2.7. Fractionation of the Inner Skin Extract by Column Chromatography

The free phenolic of inner skin extract that showed the highest total phenolic content was fractionated in a silica gel column. An amount of the extract (500 mg) was placed in a chromatographic column (20 mm diameter × 500 mm height), filled with silica gel (200–400 mesh, 60 Å), and eluted by methanol at 1 mL/min. Four fractions were obtained, based on the color of the collected fractions. All fractions were evaluated for total contents of phenolics, flavonoids, tannins, and DPPH activities. Individual phenolics were determined and quantified by high performance liquid chromatography (HPLC).

### 2.8. DPPH Radical Scavenging Activity

Free radical scavenging activity of extracts was determined by a method described previously [19]. An amount of 0.5 mL of the extracts was added to 0.25 mL of 0.5 mM DPPH and 0.5 mL of 0.1 M acetate buffer (pH 5.5), and incubated for 30 min in the dark at room temperature. The mixture absorbance was measured at 517 nm. BHT standard (10–50 ppm) was used as a positive control. The DPPH radical scavenging activity was calculated by the following Equation:

DPPH radical scavenging activity (%) = [(A_control_ − A_sample_)/A_control_] × 100

where A_control_ is the absorbance of reaction without sample, and A_sample_ is the absorbance of samples. Lower absorbance indicates a higher DPPH radical scavenging activity. IC_50_ value is defined as the concentration of the sample required to scavenge 50% of DPPH. Therefore, lower IC_50_ indicates higher antioxidant activity.

### 2.9. ABTS Radical Scavenging Activity

The assay for radical scavenging activity was carried out following an improved ABTS method, as described by Mikulic-Petkovsek et al. [20], with some adjustments. The 2,2′-azinobis (3-ethylbenzothiazoline-6-sulfonic acid) radical cation (ABTS) solution was generated by a reaction of 7 mM ABTS and 2.45 mM potassium persulfate solution (in equal quantities) after incubation at room temperature in the dark for 16 hr, and then diluted with methanol to obtain an absorbance of 0.70 ± 0.05 at 734 nm. Briefly, an aliquot of 1 mL of the ABTS solution was added to 0.120 mL of samples, and the mixture was left in the dark at room temperature for 30 min. The absorbance was recorded at 734 nm. BHT standard (0.1–0.5 mg/mL) was used as a positive control. The ABTS radical scavenging activity was calculated by the equation:

ABTS radical scavenging activity (%) = [(A_control_ − A_sample_)/A_control_] × 100

where A_control_ is the absorbance of reaction without samples and A_sample_ is the absorbance of reaction with samples. A lower absorbance therefore indicates higher ABTS radical scavenging activity. IC_50_ value was calculated as the concentration needed to scavenge 50% of ABTS. As a result, lower IC_50_ indicates higher antioxidant activity.

### 2.10. Reducing Power

Reducing power was assayed by a method described previously [21]. In brief, 1 mL extracts or BHT (0.025–0.5 mg/mL) was added in 2.5 mL phosphate buffer (0.2 M, pH 6.6) and potassium ferricyanide (2.5 mL, 1%), and then incubated at 50 °C for 30 min. Trichloroacetic acid (2.5 mL, 10%) was added to this mixture, and the mixture was centrifuged at 4000 rpm for 10 min. The supernatant (2.5 mL) was diluted with 2.5 mL of distilled water and an aliquot of 0.5 mL FeCl_3_ solution (0.1%) was added. The absorbance was measured at 700 nm. BHT standard (0.1–0.5 mg/mL) was used as a positive control. IC_50_ value was calculated at which the absorbance was 0.5, and lower IC_50_ indicates higher reducing power.

### 2.11. β-Carotene Bleaching System

The antioxidant activity of extracts was evaluated by the β-carotene linoleate bleaching system described by Deba et al. [22]. A solution of β-carotene (2 mg) was prepared in 10 mL of chloroform, and 2 mL of the chloroform solution was pipetted into a round-bottom flask with 40 µL linoleic acid and 400 mg Tween-40. The chloroform was removed under vacuum at 40 °C, and then 100 mL oxygenated water was added. The obtained emulsion was freshly prepared before each experiment. A volume of 0.12 mL of extract or BHT (1 mg/mL) was mixed with 1 mL of the emulsion. Methanol was used as a negative control. The solution was incubated at 50 °C and recorded at 492 nm. All extracts were measured at zero time and subsequently every 15 min, up to 180 min. Lipid peroxidation inhibition (LPI) was calculated using the following equation:

LPI inhibition (%) = A_1_/A_0_ × 100

where A_0_ is the absorbance value measured at zero time for the test sample, and A_1_ is the corresponding absorbance value measured after incubation for 180 min. Higher LPI value indicates stronger antioxidant activity.

### 2.12. Identification and Quantification of Phenolic Compounds by HPLC

Identification of phenolic acids and flavonoids was performed using liquid chromatography equipped with a UV detector. The phenolic acids and flavonoids were determined at 254 and 350 nm, respectively, in a JASCO HPLC system (PU-2089 Plus pump, LC-Net II/ADC controller, and UV-2075 Plus detector, Tokyo, Japan), and a column J-Pak Symphonia C18 (5 µm, 4.6 × 250 mm, 110 Å, Tokyo, Japan). A gradient elution was run with 1 mL/min flow rate using the following time gradients: 0–5 min (5% A), 5–10 min (20% A), 10–20 min (50% A), 20–30 min (80% A), 30–50 min (100% A), 50–60 min (5% A). Solvent A was 100% methanol and solvent B was water with 0.1% acetic acid. Phenolic acid and flavonoid standards (10–100 ppm) were used to establish calibration curves, and extracts (1 mg/mL) were injected with an amount of 5 µL.

### 2.13. Statistical Analysis

Data were analyzed by one-way ANOVA using the Minitab 16.0 software (Minitab Inc., State College, PA, USA). Pearson correlation coefficients were calculated by the SPSS 20 software (SPSS Inc., Chicago, IL, USA). The 1/IC_50_ values were used to indicate Pearson correlation coefficients (*R*) between total phenolic, flavonoid and tannin contents and antioxidant activities. Upon significant differences, means were separated using Tukey’s test at *p* < 0.05 with three replications and expressed as the mean ± standard errors (SE).

## 3. Results

### 3.1. Total Phenolic, Flavonoid and Tannin Contents

**Figure 1:**
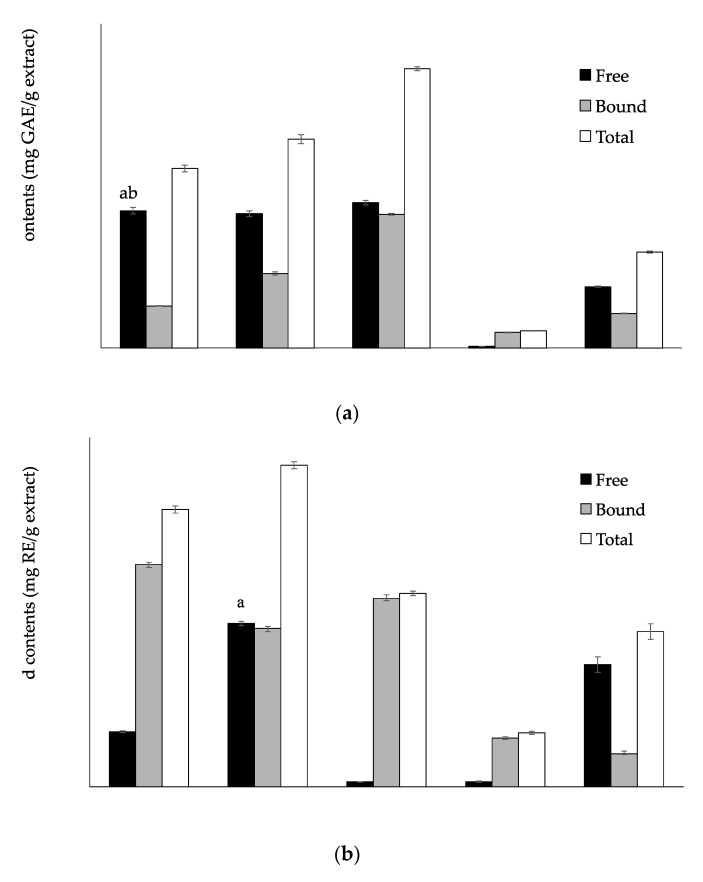

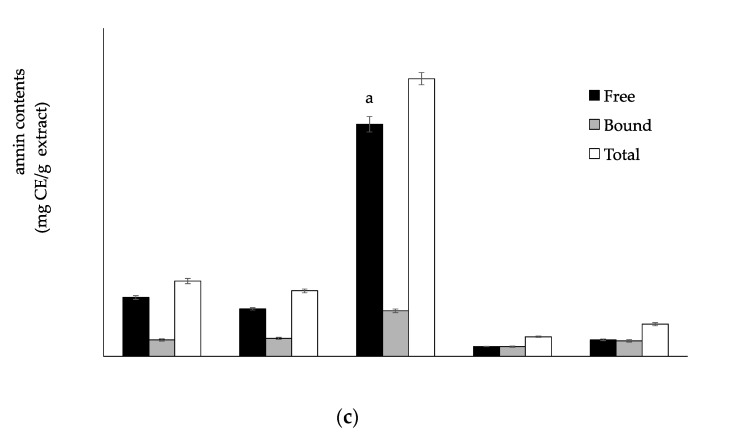
Phenolic (**a**), flavonoid (**b**), and tannin contents (**c**) of free and bound phenolic extracts and their total contents in *C. crenata.* Different letters within the same color columns indicate significant differences (*p* < 0.05).

Phenolic, flavonoid and tannin contents are given in Figure 1a–c, respectively. The free and bound phenolic contents of inner skin extract had the highest values, which resulted in maximum total phenolic contents (1034 ± 7.21 mg GAE/g extract) (*p* < 0.05) (Figure 1a). On the other hand, flowers showed the greatest quantity of total flavonoids (147.41 ± 1.61 mg RE/ g extract), followed by barks and inner skins (Figure 1b). Accordingly, the inner skins showed significantly higher amounts of total tannins (253.89 ± 5.59 mg CE/g extract) (Figure 1c). Therefore, the free extract of inner skins was selected for further analysis.

### 3.2. Antioxidant Activity

The antioxidant activities of extracts compared with standard BHT are listed in Table 1. For free phenolic extracts, inner skin extract showed the highest radical scavenging activity and reducing power. The antioxidant activity of inner skin extract in DPPH assay was maximum (23.81 ± 0.07 µg/mL) and it was greater than the standard compound BHT (27.27 ± 0.53 µg/mL) (*p* < 0.05). Flower and bark extracts were also indicated to have promising antioxidant activity. Except for kernels, all of the extracts showed strong bleaching inhibition, and no significant difference to BHT activity was observed (*p* > 0.05) (Table 1). Regarding to the bound phenolic extracts, the inner skins also showed maximum antioxidant capacities. IC_50_ values of DPPH (28.41 ± 0.25 µg/mL) and reducing power (209.56 ± 0.62 µg/mL) were the highest, and not significantly different to that of BHT (*p* > 0.05) (Table 1).

**Figure 2:**
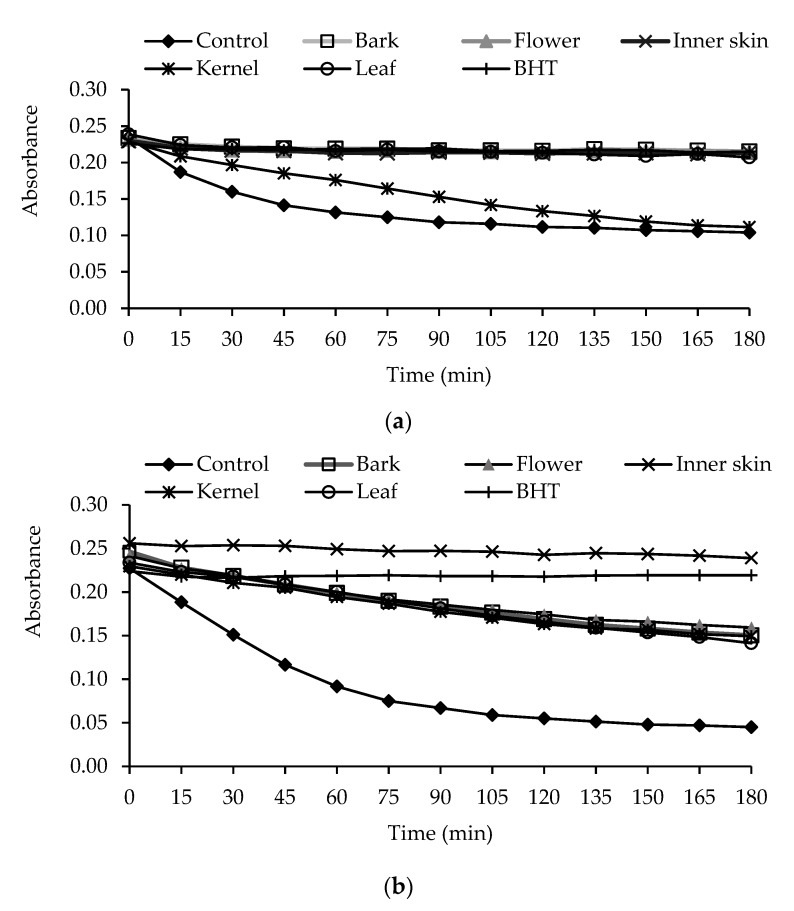
Antioxidant activity of free phenolic (**a**) and bound phenolic extracts (**b**) of *C. crenata* measured by β-carotene bleaching method.

The percentage of lipid peroxidation inhibition of extracts is presented in Figure 2. In free phenolic extracts, leaves, barks, flowers, and inner skins showed high capacities to inhibit β-carotene bleaching, and they were at similar levels to BHT (Figure 2a). Kernels also inhibited β-carotene bleaching, but had lower antioxidant properties than the other plant parts. In bound phenolic extracts, inner skins showed the greatest activity, as compared to BHT and other plant parts (Figure 2b). The percentage of lipid peroxidation inhibition (LPI %) of inner skin extract was also the strongest. In general, the free phenolic extracts possessed greater antioxidant capacity than the bound phenolic extracts (Table 1).

### 3.3. Correlations between Antioxidant Activity and Phenolic Contents

Table 2 demonstrated a significantly strong and positive correlation between the phenolic contents and antioxidant activities of extracts in all tested assays, especially in ABTS (*R* = 0.91) and reducing power (*R* = 0.99). Similarly, the tannin contents had a highly positive correlation to antioxidant activities (*R* = 0.47–0.64). In contrast, the flavonoid contents did not correlate to antioxidant activity of the extracts. It was found that the phenolic and tannin contents were proportional to the antioxidant capacity of *C. crenata*.

### 3.4. Identification of Individual Phenolic Acids and Flavonoids of C. crenata

**Figure 3:**
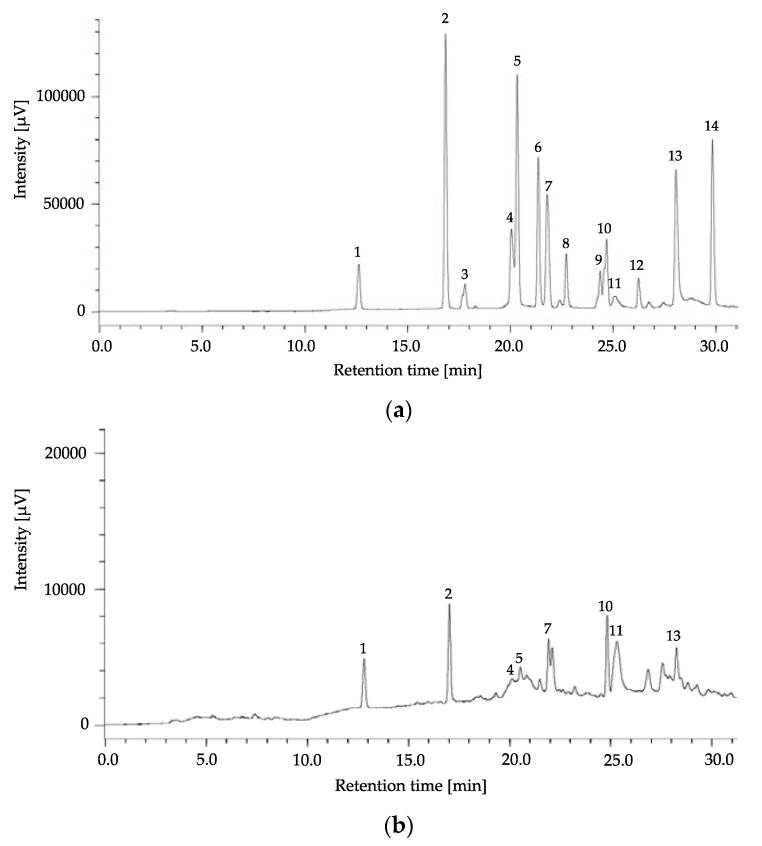
High performance liquid chromatography (HPLC) chromatograms of phenolic standard mixture (**a**) and bound phenolics of bark extract (**b**). Peak 1: gallic acid; 2: protocatechuic acid; 3: catechol; 4: chlorogenic acid; 5: *p*-hydroxybenzoic acid; 6: vanillic acid; 7: syringic acid; 8: vanillin; 9: ferulic acid; 10: sinapic acid; 11: *p*-coumaric acid; 12: benzoic acid; 13: ellagic acid; 14: cinnamic acid.

Eight free phenolic acids consisting of gallic, protocatechuic, sinapic, *p*-coumaric, benzoic, ellagic acids, catechol, and vanillin were identified (Table 3). Free ellagic acid was the most abundant phenolic (3.11 mg/g dry weight in total), followed by free *p*-coumaric acid (3.07 mg/g dry weight in total). Free vanillin and benzoic acid were solely detected in bark and flower extracts, respectively. Six free phenolic acids were found in flower extract. The number of phenolic acids detected in bound extracts was greater than free extracts. The highest number of phenolics, eight compounds, were accumulated in the bound extracts of barks and leaves (Figure 3); whereas the lowest number of phenolics, five compounds, were detected in kernels. Concentrations of the detected phenolic acids were variable among plant parts. Accordingly, the inner skin extract obtained a maximum amount of gallic and *p*-coumaric acids with 5.20 ± 0.12 and 2.61 ± 0.15 mg/g dry weight, respectively (*p* < 0.05).

**Figure 4:**
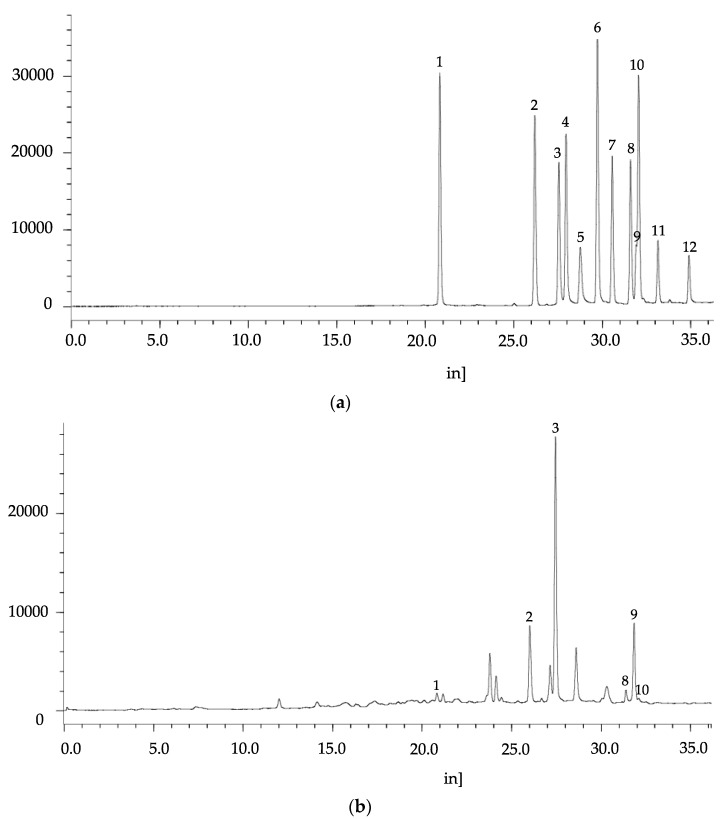
HPLC chromatograms of flavonoid standard mixture (**a**) and bound flavonoids of flower extract (**b**). Peak 1: esculetin; 2: isoquercitin; 3: myricetin, 4: fisetin; 5: morin; 6: quercetin; 7: luteolin; 8: kaemferol; 9: isohamnetin; 10: apigenin; 11: rhamnetin; 12: galangin.

The identified flavonoids are shown in Table 4 and Figure 4. Myricetin and isoquercitrin were predominant compounds, and they were available in both free and bound extracts. Esculetin, morin, and apigenin were presented only in bound extracts, while fisetin and rhamnetin were solely detected in free extracts.

### 3.5. Fractionation of Free Phenolic Extract of Inner Skins by Column Chromatography

Total phenolic and tannin contents with the relevant DPPH scavenging activity of four fractions of inner skin extract are displayed in Table 5. Factions 1 (F1) and 3 (F3) showed the greatest total phenolic contents. The maximum total tannin contents were shown in F3 which were three-fold higher than F4. Meanwhile, F4 contains the highest total flavonoid content. Fractions F1, F2, and F3 with IC_50_ < 11.56 µg/mL exhibited stronger antioxidant properties than the inner skin crude extract and BHT (Table 1). Gallic, protocatechuic, *p*-coumaric, and ellagic acids were detected by HPLC analysis. Of these, gallic acid was the principal compound, whereas *p*-coumaric and ellagic acids were found only in the F3 fraction.

## 4. Discussion

Chestnut (*C. crenata*) has valuable pharmaceutical properties to human health, as its leaves, flowers, nuts, and inner skins contain abundant phenols, and exhibit strong antioxidant activity [2,10,23,24]. Comparatively, the total phenolic contents of flowers, inner skins, kernels, and leaves of *C. crenata* were considerably higher than *Castanea sativa* Miller (3.73–475 mg GAE/g extract) [25]. Total phenolic and flavonoid contents of inner skins were also greater than previous results reported by Lee et al. [24] that *C. crenata* possessed only 5.33 mg GAE/g in total phenolic contents, and 0.12 mg QE/g in total flavonoid contents. Phenolic acids and tannins are often stored in plant seeds to protect from pathogen and insect attack [26]; in agreement, inner skins were found to obtain high contents of these compounds in this study.

Antioxidant activity is one common parameter to estimate the quality and function of bioactive constituents in foods and pharmaceutics [21]. DPPH and ABTS radical scavenging methods are typically spectrophotometric procedures, and are widely used to determine the antioxidant properties of extracts. Those methods are based on the reductions of DPPH and ABTS in methanol solution, in the presence of hydrogen-donating antioxidants [27]. Regarding the reducing power assay, the reduction capacity of a compound may serve as an indicator of its potential antioxidant activity [27]. In this method, an increase in the absorbance indicates stronger antioxidant activity. β-carotene is also a useful method to measure the antioxidant activity of plant extracts, because it is extremely susceptible to the free-radical-mediated oxidation of linoleic acid [28].

The presence and quantity of phenolic compounds and antioxidant capacity are correlated with each other for their activities [29]. In this study, the total phenolic contents exhibited a strong correlation with antioxidant activities such as DPPH and ABTS scavenging, reducing power as well as lipid peroxidation inhibition (*R* > 0.64) (Table 2), and this result was supported by the study by Ayoub et al. [7]. Phenolics were reported to play an important role in antioxidant activity, and higher phenolic contents lead to stronger antioxidant activity [30].

Identification and quantification of phenolic acids and flavonoids were carried out by comparing retention times and peak areas of the standard compounds analyzed under the same experimental conditions. This study is the first to reveal the profiles of phenolic acids and flavonoids in *C. crenata*. Gallic, ellagic, chlorogenic, and *p*-coumaric acids quantified in bark, flower, inner skin, and leaf extracts of *C. crenata* were present in greater amounts than that of kernel extracts of *Castanea sativa* [31]. Gallic acid, ellagic acid, myricetin, and isoquercitrin were the principal components in barks, flowers, inner skins, and leaves of *C. crenata*. Structurally, ellagic acid includes four hydroxyl groups bonded to the aromatic ring, which is the most important factor associated with strong DPPH scavenging activity [32]. Previously, ellagic acid was referred to inhibit lipid peroxidation, and it also possessed much stronger radical scavenging activity than dl-α-tocopherol [13]. Therefore, bark, flower, inner skin, and leaf extracts showed very high LPI values (Table 2), that may be due to the presence of ellagic acid. The fractions F1 and F3 exhibited the highest activity in the DPPH assay, and contained gallic and *p*-coumaric acids (Table 5).

Phenolics and flavonoids are considered to be important compounds in the human diet. The estimated range of consumption is 25–1000 mg a day for phenolics and 50–800 mg for flavonoids [33]. These compounds have been reported to reduce the risk of metabolic syndromes, and the related complications of Type 2 diabetes, and phenolic constituents could potentially yield benefits for human health [34]. Flavonoids have also been used as treatments for ovarian, breast cervical, pancreatic, and prostate cancer [35]. Gallic and protocatechuic acids have activities that inhibit α-glucosidase and α-amylase, which are key enzymes for the digestion of dietary carbohydrates [36]. Ellagic acid, a powerful bioactive compound, possesses antioxidative, anticarcinogenic, and anti-inflammatory activities, and has been used for the treatment of either several types of cancers, or cardiovascular, parasitic, eye, and kidney diseases [37].

## 5. Conclusions

This paper provides a strong evidence for the antioxidant activities and free and bound phenolic profiles of *C. crenata*. The work herein indicates that bark, flower, and inner skin extracts showed potent antioxidant activity in vitro, while inner skins had the best antioxidant power in both free and bound phenolic extracts. After fractionation of inner skins by column chromatography, three fractions showed higher antioxidant activity than free phenolic extract. In comparison with previous studies, this work profiles 13 phenolic acids and eight flavonoids of *C. crenata* for the first time. *C. crenata* is a promising natural preservative agent with potential applications in food and pharmaceutical industries.

## Figures and Tables

**Table 1:** Antioxidant activities of free and bound phenolic extracts.

Antioxidant Activities	Bark	Flower	Inner Skin	Kernel	Leaf	BHT
*Free phenolic extract*						
DPPH (IC_50_ μg/mL)	25.59 ± 0.22 ^c,d^	29.45 ± 0.22 ^b^	23.81 ± 0.07 ^d^	NA	48.98 ± 0.61 ^a^	27.27 ± 0.53 ^c^
ABTS (IC_50_ μg/mL)	300.26 ± 3.22 ^c^	289.45 ± 1.30 ^c,d^	270.73 ± 5.36 ^d^	>10,000 ^a^	602.20 ± 9.35 ^b^	184.18 ± 3.01 ^e^
Reducing power (IC_50_ μg/mL)	186.07 ± 0.91 ^d^	197.50 ± 1.04 ^c^	187.78 ± 1.88 ^c,d^	>10,000 ^a^	435.72 ± 4.85 ^b^	185.23 ± 1.46 ^d^
Bleaching inhibition (LPI %)	92.16 ± 0.74 ^a^	92.79 ± 1.02 ^a^	93.03 ± 0.06 ^a^	48.63 ± 9.38 ^b^	86.77 ± 3.23 ^a^	95.02 ± 2.28 ^a^
*Bound phenolic extract*						
DPPH (IC_50_ μg/mL)	103.50 ± 1.70 ^c^	49.43 ± 0.17 ^d^	28.41 ± 0.25 ^e^	1680 ± 8.67 ^a^	467.93 ± 1.39 ^b^	27.27 ± 0.53 ^e^
ABTS (IC_50_ μg/mL)	809.64 ± 0.00 ^c^	386.46 ± 1.88 ^e^	538.70 ± 13.30 ^d^	>4000 ^a^	3193 ± 18.00 ^b^	184.18 ± 3.01 ^f^
Reducing power (IC_50_ μg/mL)	726.00 ± 10.10 ^c^	344.85 ± 3.28 ^d^	209.56 ± 0.62 ^e^	3944 ± 29.10 ^a^	2542 ± 57.50 ^b^	185.23 ± 1.46 ^e^
Bleaching inhibition (LPI %)	61.02 ± 0.74 ^b^	66.02 ± 1.14 ^b^	93.35 ± 2.76 ^a^	64.96 ± 5.11 ^b^	60.45 ± 6.75 ^b^	97.92 ± 1.04 ^a^

NA: None active. The data represent the means ± SE (*n* = 3). (^a–f^) Different letters in each row indicate significant difference (*p* < 0.05). BHT (Dibutyl hydroxytoluene) is the positive control. DPPH: 1,1-diphenyl-2-picrylhydrazyl, ABTS: 2,2′-azinobis (3-ethylbenzothiazoline-6-sulfonic acid) diammonium salt, LPI: Lipid peroxidation inhibition.

**Table 2:** Pearson correlation coefficients (*R*) between total phenolic, flavonoid, and tannin contents and antioxidant activities.

Correlated Components	TPC	TFC	TTC	DPPH (1/IC_50_)	ABTS (1/IC_50_)	Reducing Power (1/IC_50_)
TFC	0.20					
TTC	0.64 **	−0.34				
DPPH (1/IC_50_)	0.64 **	0.04	0.56 **			
ABTS (1/IC_50_)	0.91 **	0.18	0.64 **	0.88 **		
Reducing Power (1/IC_50_)	0.99 **	0.21	0.61 **	0.67 **	0.94 **	
LPI	0.71 **	0.01	0.47 **	0.45 *	0.66 **	0.71 **

**, *: Correlation is significant at the 0.01 and 0.05 levels, respectively. TPC: total phenolic content; TFC: Total flavonoid content; TTC: Total tannin content.

**Table 3:** Profile of identified phenolic acids from *C. crenata*.

Phenolic Acids (mg/g Dry Weight)	Retention Time (min)	Bark	Flower	Inner Skin	Kernel	Leaf	Total
*Free phenolic acids*							
Gallic acid	12.80	0.14 ± 0.01 ^b^	1.71 ± 0.05 ^a^	-	-	-	1.85
Protocatechuic acid	16.83	0.13 ± 0.00 ^b^	0.41 ± 0.05 ^a^	-	-	-	0.54
Catechol	17.78	-	1.11 ± 0.04	-	-	-	1.11
Vanillin	22.70	0.68 ± 10.09	-	-	-	-	0.68
Sinapic acid	24.81	-	0.89 ± 0.00	-	-	-	0.89
*p*-Coumaric	25.20	0.46 ± 0.00 ^b^	-	2.61 ± 0.15 ^a^			3.07
Benzoic acid	26.29	-	0.65 ± 0.12	-	-	-	0.65
Ellagic acid	28.35	1.49 ± 0.06 ^a^	0.71 ± 0.02 ^b^	0.51 ± 0.01 ^c^	-	0.40 ± 0.01 ^d^	3.11
Total		2.90	5.48	3.12		0.40	
*Bound phenolic acids*							
Gallic acid	12.80	0.29 ± 0.01 ^c^	2.22 ± 0.01 ^b^	5.20 ± 0.12 ^a^	-	-	7.71
Protocatechuic acid	16.83	0.35 ± 0.01 ^a^	0.15 ± 0.00 ^b^	0.39 ± 0.00 ^a^	0.01 ± 0.00 ^c^		0.90
Chlorogenic acid	20.05	0.94 ± 0.04 ^a^	-	-	-	0.82 ± 0.00 ^a^	1.76
*p*-Hydroxybenzoic	20.60	0.08 ± 0.01 ^b^	0.06 ± 0.01 ^b^	0.14 ± 0.02 ^a^	0.01 ± 0.00 ^c^	0.05 ± 0.00 ^b^	0.34
Vanillic acid	21.58	-	0.05 ± 0.01 ^a^	-	-	0.04 ± 0.00 ^a^	0.09
Syringic acid	22.02	1.71 ± 0.06	-	-	-	-	1.71
Ferulic acid	24.50	-	0.52 ± 0.00 ^a^	-	0.01 ± 0.00 ^c^	0.21 ± 0.01 ^b^	0.74
Sinapic acid	24.81	1.74 ± 0.03 ^a^	0.69 ± 0.01 ^c^	-	0.05 ± 0.00 ^d^	1.06 ± 0.00 ^b^	3.54
*p*-Coumaric	25.20	0.98 ± 0.07 ^a^	-	-	0.02 ± 0.00 ^c^	0.36 ± 0.05 ^b^	1.36
Benzoic acid	26.29	-	-	-	-	0.76 ± 0.00	0.76
Ellagic acid	28.35	0.32 ± 0.01 ^b^	0.94 ± 0.01 ^a^	0.33 ± 0.01 ^b^	-	0.09 ± 0.01 ^c^	1.68
Total		6.41	4.63	6.06	0.10	3.39	

The data represent the means ± SE (*n* = 4). (^a–d^) Different letters in each row indicate significant difference (*p* < 0.05). (-) Not detected.

**Table 4:** Profile of identified flavonoids from *C. crenata*.

Flavonoids (mg/g Dry Weight)	Retention Time (min)	Bark	Flower	Inner Skin	Kernel	Leaf	Total
*Free flavonoids*							
Isoquercitrin	26.18	-	1.43 ± 0.01 ^b^	-	-	3.96 ± 0.04 ^a^	5.39
Myricetin	27.53	1.16 ± 0.10 ^a^	0.49 ± 0.09 ^b^	-	-	0.54 ± 0.10 ^b^	2.19
Fisetin	27.93	0.60 ± 0.00 ^b^	-	-	-	1.37 ± 0.01 ^a^	1.97
Kaempferol	31.58	-	-	-	-	0.18 ± 0.02	0.18
Rhamnetin	33.13	-	0.49 ± 0.00	-	-	-	0.49
Total		1.76	2.41	-	-	6.05	
*Bound flavonoids*							
Esculetin	20.85	-	0.16 ± 0.01 ^b^	0.61 ± 0.01 ^a^	-	-	0.77
Isoquercitrin	26.18	-	0.69 ± 0.01	-	-	-	0.69
Myricetin	27.53	0.10 ± 0.03 ^c^	3.59 ± 0.07 ^a^	-	-	2.13 ± 0.07 ^b^	5.82
Morin	28.79	0.37 ± 0.03 ^b^	0.17 ± 0.00 ^b^	1.22 ± 0.08 ^a^	-	-	1.76
Kaempferol	31.58	-	0.09 ± 0.00	-	-	-	0.09
Apigenin	32.08	-	-	1.04 ± 0.22	-	-	1.04
Total		0.47	4.70	2.87	-	2.13	

The data represent the means ± SE (*n* = 4). (^a–c^) Different letters in each row indicate significant differences (*p* < 0.05). (-) Not detected.

**Table 5:** Total contents of polyphenols and phenolic acids (mg/g extract), and radical scavenging activity (µg/mL) of inner skin fractionations.

Fractions	TPC	TFC	TTC	DPPH (IC_50_)	Gallic Acid	Protocatechuic Acid	*p*-Coumaric Acid	Ellagic Acid
F1	482.71 ± 19.41 ^a^	7.44 ± 0.19 ^c^	58.11 ± 1.91 ^a^	6.70 ± 0.09 ^c^	4.27 ± 0.53 ^a^	0.30 ± 0.01	-	-
F2	404.03 ± 2.04 ^b^	6.93 ± 0.13 ^c^	59.83 ± 0.82 ^a^	11.56 ± 0.21 ^b^	3.74 ± 0.00 ^a,b^	-	-	-
F3	514.22 ± 8.51 ^a^	16.07 ± 0.55 ^b^	60.50 ± 1.27 ^a^	5.71 ± 0.26 ^c^	5.17 ± 0.45 ^a^	-	19.85 ± 2.96	4.12 ± 0.08
F4	210.67 ± 0.77 ^c^	22.85 ± 0.55 ^a^	20.17 ± 0.75 ^b^	29.09 ± 0.62 ^a^	1.78 ± 0.09 ^b^	-	-	-

The data represent the means ± SE (*n* = 3). (^a–c^) Different letters in each column indicate significant difference (*p* < 0.05); TPC: Total phenolic content; TFC: Total flavonoid content; TTC: Total tannin content.
